# Supplementary material for: The Systems Biology Research Tool: evolvable open-source software
Source: BMC Syst Biol. 2008 Jun 29;2:55. doi: 10.1186/1752-0509-2-55 (PMC2446383; doi:10.1186/1752-0509-2-55)
Supplement: Additional file 1 — SBRT Archive. An archive of the current version of the Systems Biology Research Tool. [file 1752-0509-2-55-S1.zip › sbrt-1.4.0/doc/developers_guide/api/sbrt/shell/text/math/CoeffFormatV2.html]

CoeffFormatV2


|  |  |  |
| --- | --- | --- |
| **Overview** \| **Package** \| **Class** \| **Use** \| **Tree** \| **Deprecated** \| **Index** \| **Help** | |  |
| **PREV CLASS**   **NEXT CLASS** | **FRAMES**    **NO FRAMES**     **All Classes** |  |
| SUMMARY: NESTED \| FIELD \| CONSTR \| METHOD | DETAIL: FIELD \| CONSTR \| METHOD |  |


---


## sbrt.shell.text.math Class CoeffFormatV2

```
java.lang.Object
  sbrt.shell.text.math.CoeffFormatV2
```

**All Implemented Interfaces:**: Format, Formatter<java.lang.Double>, CoeffFormat, Parser<java.lang.Double>, SimpleFormat<java.lang.Double>

---

``` public final class CoeffFormatV2 extends java.lang.Object implements CoeffFormat ```

This class is a concrete implementation of
`CoeffFormat`. The string representation of
a coefficient is: *numerical value*.

**Author:**
:   This class was written and documented by
    Jeremiah Wright while in the Wagner lab.

---

**Constructor Summary**

`CoeffFormatV2()`
  
          Constructs a new coefficient format object.


| **Method Summary** | |
| --- | --- |
| `java.lang.String` | `format(double coefficient)`             Returns a formatted string representation of the provided coefficient. |
| `java.lang.String` | `format(java.lang.Double coefficient)`             Returns a formatted string representation of the provided coefficient. |
| `DoubleFormatV1` | `getNumberFormat()`             Returns the format used for numerical values. |
| `java.lang.Double` | `parse(java.lang.String coefficient)`             Parses the provided string and returns its corresponding coefficient. |
| `double` | `parsePrimitive(java.lang.String coeff)`             Parses the provided string and returns its corresponding coefficient. |

**Methods inherited from class java.lang.Object**

`clone, equals, finalize, getClass, hashCode, notify, notifyAll, toString, wait, wait, wait`

**Constructor Detail**

### CoeffFormatV2

```
public CoeffFormatV2()
```

:   Constructs a new coefficient format object.


**Method Detail**

### getNumberFormat

```
public DoubleFormatV1 getNumberFormat()
```

:   Returns the format used for numerical values.

    :   **Returns:**: the format used for numerical values.

---


### format

```
public java.lang.String format(java.lang.Double coefficient)
```

:   Returns a formatted string representation of the
    provided coefficient.

    :   **Specified by:**: `format` in interface `Formatter<java.lang.Double>` **Specified by:**: `format` in interface `CoeffFormat`
    :   **Parameters:**: `coefficient` - the coefficient to be formatted. **Returns:**: a formatted string representation of the provided coefficient. **Throws:**: `java.lang.NullPointerException` - if the provided coefficient is `null`.

---


### format

```
public java.lang.String format(double coefficient)
```

:   Returns a formatted string representation of the
    provided coefficient.

    :   **Specified by:**: `format` in interface `CoeffFormat`
    :   **Parameters:**: `coefficient` - the coefficient to be formatted. **Returns:**: a formatted string representation of the provided coefficient. **Throws:**: `java.lang.NullPointerException` - if the provided coefficient is `null`.

---


### parse

```
public java.lang.Double parse(java.lang.String coefficient)
```

:   Parses the provided string and returns its
    corresponding coefficient.

    :   **Specified by:**: `parse` in interface `CoeffFormat` **Specified by:**: `parse` in interface `Parser<java.lang.Double>`
    :   **Parameters:**: `coefficient` - the string to be parsed. **Returns:**: the coefficient corresponding to the provided string. **Throws:**: `java.lang.NullPointerException` - if the provided coefficient is `null`.

---


### parsePrimitive

```
public double parsePrimitive(java.lang.String coeff)
```

:   Parses the provided string and returns its
    corresponding coefficient.

    :   **Specified by:**: `parsePrimitive` in interface `CoeffFormat`
    :   **Parameters:**: `coeff` - the string to be parsed. **Returns:**: the coefficient corresponding to the provided string. **Throws:**: `java.lang.NullPointerException` - if the provided coefficient is `null`.


---


|  |  |  |
| --- | --- | --- |
| **Overview** \| **Package** \| **Class** \| **Use** \| **Tree** \| **Deprecated** \| **Index** \| **Help** | |  |
| **PREV CLASS**   **NEXT CLASS** | **FRAMES**    **NO FRAMES**     **All Classes** |  |
| SUMMARY: NESTED \| FIELD \| CONSTR \| METHOD | DETAIL: FIELD \| CONSTR \| METHOD |  |


---
